# Body mass index, genetic susceptibility, and Alzheimer's disease: a longitudinal study based on 475,813 participants from the UK Biobank

**DOI:** 10.1186/s12967-022-03621-2

**Published:** 2022-09-09

**Authors:** Shiqi Yuan, Wentao Wu, Wen Ma, Xiaxuan Huang, Tao Huang, MIn Peng, Anding Xu, Jun Lyu

**Affiliations:** 1 Department of Neurology, The First Affiliated Hospital of Jinan University, No. 613, Huangpu Road West, Guangzhou, 510630 Guangdong China; 2 School of Public Health, Jiaotong University Health Science Center, Shaanxi Province, Xi’an, 710061 China; 3 Department of Clinical Research, The First Affiliated Hospital of Jinan University, No. 613, Huangpu Road West, Guangzhou, 510630 Guangdong China; 4 Guangdong Provincial Key Laboratory of Traditional Chinese Medicine Informatization, Guangzhou, Guangdong China

**Keywords:** Body mass index (BMI), Genetic susceptibility, Alzheimer's disease (AD), Bidirectional Mendelian randomization (MR), UK Biobank

## Abstract

**Background:**

The association between body mass index (BMI) and Alzheimer's disease (AD) remains controversial. Genetic and environmental factors are now considered contributors to AD risk. However, little is known about the potential interaction between genetic risk and BMI on AD risk.

**Objective:**

To study the causal relationship between BMI and AD, and the potential interaction between AD genetic risk and BMI on AD risk.

**Methods and Results:**

Using the UK Biobank database, 475,813 participants were selected for an average follow-up time of more than 10 years. Main findings: 1) there was a nonlinear relationship between BMI and AD risk in participants aged 60 years or older (*p* for non-linear < 0.001), but not in participants aged 37–59 years (*p* for non-linear = 0.717) using restricted cubic splines; 2) for participants aged 60 years and older, compared with the BMI (23–30 kg/m^2^) group, the BMI (< 23 kg/m^2^) group was associated with a higher AD risk (HR = 1.585; 95% CI 1.304–1.928, *p* < 0.001) and the BMI (> 30 kg/m^2^) group was associated with a lower AD risk (HR = 0.741; 95% CI 0.618–0.888, *p* < 0.01) analyzed using the Cox proportional risk model; 3) participants with a combination of high AD genetic risk score (AD-GRS) and BMI (< 23 kg/m^2^) were associated with the highest AD risk (HR = 3.034; 95% CI 2.057–4.477, *p* < 0.001). In addition, compared with the BMI (< 23 kg/m^2^), the higher BMI was associated with a lower risk of AD in participants with the same intermediate or high AD-GRS; 4) there was a reverse causality between BMI and AD when analyzed using bidirectional Mendelian randomization (MR).

**Conclusion:**

There was a reverse causality between BMI and AD analyzed using MR. For participants aged 60 years and older, the higher BMI was associated with a lower risk of AD in participants with the same intermediate or high AD genetic risk. BMI (23–30 kg/m^2^) may be a potential intervention for AD.

**Supplementary Information:**

The online version contains supplementary material available at 10.1186/s12967-022-03621-2.

## Background

Dementias are chronic, progressive neurological diseases characterized by memory loss and cognitive impairment [1]. Among the dementias, the most common one is Alzheimer's disease (AD), which accounts for approximately 50–70% of all dementia patients [2]. Critically, its prevalence is rising sharply, owing to the global population aging [3]. Therefore, reducing the burden of AD has become an important global public health issue [4].

The main pathological features of AD include amyloid plaques and neuronal filament entanglement [5, 6]. AD is believed to arise from a combination of genetic and environmental factors and it can be divided into early-onset AD (EOAD) and late-onset AD (LOAD) [3, 7]. It is important to identify the gene-environment interactions behind the development of AD, which would allow for the development of personalized intervention strategies for early intervention of AD, thereby ultimately reducing the global incidence of AD [8].

In conjunction with the rising rate of AD, there is also a worrying epidemic of high levels of obesity worldwide [9]. As an indicator of body nutrition, body mass index (BMI) has been reported to be associated with cerebrovascular adverse events, a variety of cancers, and other diseases [10–13]. However, the association between BMI and AD risk remains controversial [14]. Several studies have shown that obesity and weight loss in middle age are associated with an increased risk of dementia [15, 16], while other studies have shown that obesity in old age does not increase the risk of AD [16, 17]. Moreover, a large UK population study has shown that low BMI across all age groups increases the risk of AD [18]. Although many previous studies have focused on the association between BMI and AD, these conflicting results suggest that the causal relationship between BMI and AD requires further exploration. Moreover, gene-environment interactions behind the development of AD also need deep investigation. However, little is known about the potential interaction between genetic risk and BMI on AD risk so far.

Understanding the causal relationship between BMI and AD is crucial for AD prevention. However, simple observational studies tend to result in reverse causality and residual confusion [19]. Mendelian randomization (MR) based on genetic variations is useful to overcome some of these limitations [20]. Numerous previous studies using MR analysis to assess the causal effect of BMI on AD found that polygenic scores strongly related to a higher BMI are unrelated to higher dementia risk and may even predict a lower dementia risk. This is surprising, however, there has been no further assessment of reverse causality [21]. Fortunately, bidirectional MR overcomes this limitation [22]. For the first time, this study used bidirectional MR to assess the causal relationship between BMI and AD. In addition, since we conducted observational studies and MR in the same study population, the conclusions could be more stable and reliable.

Therefore, we sought to use the UK Biobank (UKB) to investigate the relationship between BMI and risk of developing AD. To further assess the relationship between BMI and genetic susceptibility on AD risk, we also explored potential genetic and BMI interactions after calculating the AD genetic risk score (AD-GRS) of each participant. Finally, bidirectional MR was used to further explore the causal relationship between BMI and AD.

## Methods

### Study population

A public database is specifically designed to store scientific research data on an open platform [23]. The UK Biobank (UKB) is the world's largest biomedical sample database and contains data from a population-based cohort study consisting of more than 500,000 volunteers. The UKB study was approved by the Northwest Multicenter Research Ethics Committee, and all participants agreed to their inclusion [24, 25]. Importantly, it has collected—and continues to collect—a large number of participant data regarding phenotypes and genotypes [26, 27].

**Fig. 1:**
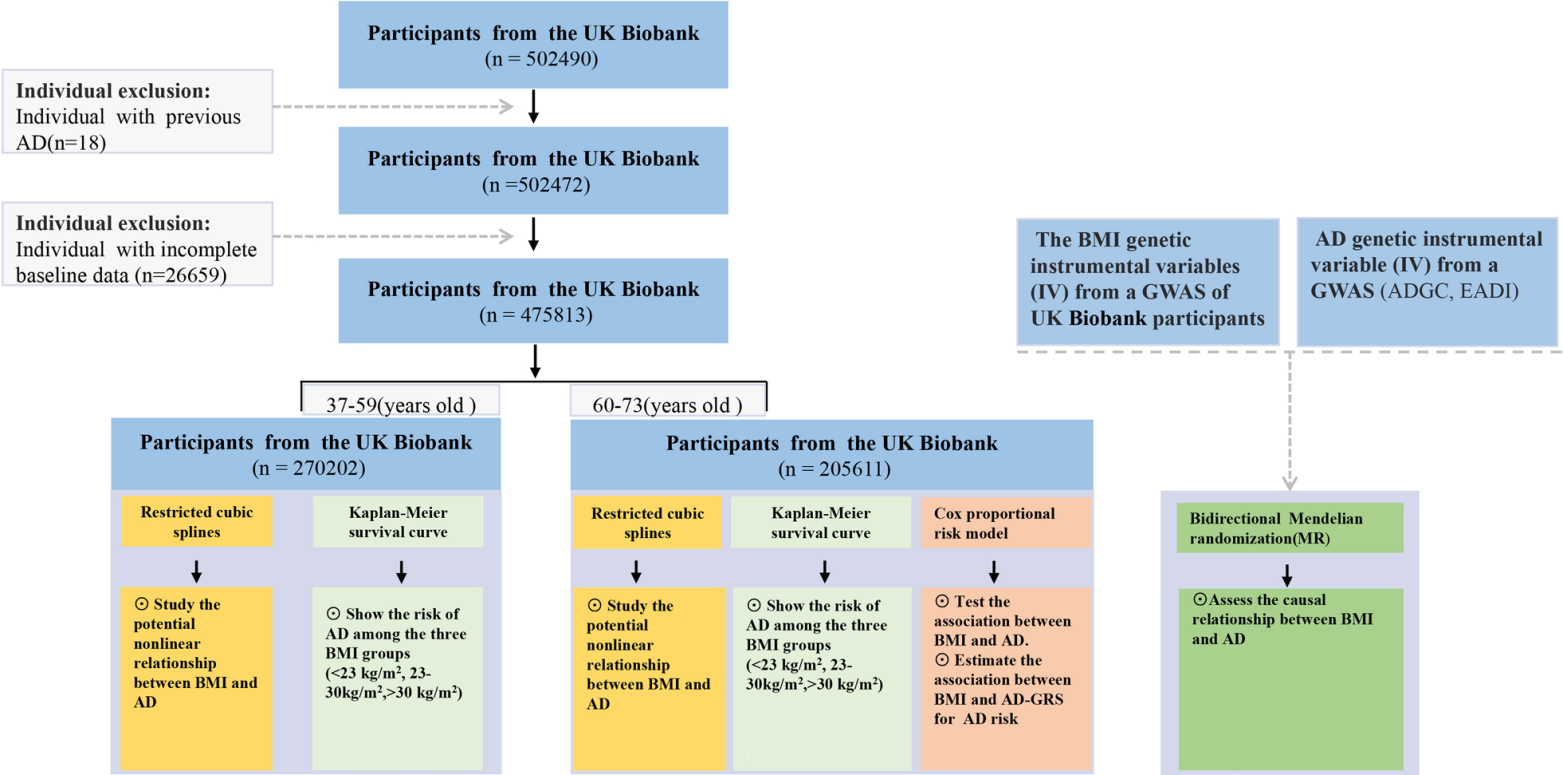
The flow chart of the analysis process. BMI, Body mass index; AD, Alzheimer's disease; AD-GRS, Alzheimer's disease genetic risk score; ADGC, Alzheimer Disease Genetics Consortium; EADI, European Alzheimer's Disease Initiative; MR, Mendelian randomization

Initially, 502,490 participants were enrolled, excluding the participants who.

had been diagnosed with AD prior to registration (n = 18) and who either did not undergo genetic testing (n = 13,121) or did not complete baseline data collection (n = 26,659). This resulted in 475,813 participants who were enrolled in our study. A study flow chart of the analysis process is presented in Fig. 1. All participants have a complete case analysis.

### Ascertainment of exposure and basic characteristics

According to the UKB, BMI was measured by weight (kg) divided by height measured per square meter (m^2^). The basic characteristics of each participant were primarily identified using registration records. Follow-up occurred from the registration date to the time of AD diagnosis, death, or final follow-up time (December 2020), whichever occurred first.

### Definition of genetic risk score

Regarding quality control, the input procedures and genotyping for participants included in the UKB have been described previously [28]. In this study, the newly discovered loci from the UKB were not included to reduce false positives. 29 independent single nucleotide polymorphisms (SNPs) with significant association with AD were selected based on a previous genome-wide association study (GWASs) [29–31].

The selected SNPs are listed in the Additional file 2: Table S1. For each individual included in the UKB, an AD-GRS was determined using the previously published method [32]. The effect size (beta-coefficient) of each SNP was derived from the reported GWAS results [30]. Each participant was assigned as one of the following genetic risks for AD: High (5 quintile), intermediate (2–4 quartile), or low (1 quintile).

### Outcome assessment

In the UKB database, the AD outcome of each participant is determined by algorithmically-defined outcomes (https://www.ukbiobank.ac.uk).

### MR analyses

MR is based on the natural and random classification of genetic variation during meiosis to produce the random distribution of genetic variation in a population [20]. Genetic variation has been used as an instrumental variable (IV). In bidirectional MR, exposure instruments and outcome are used to assess whether the “exposure” variable causes the “outcome” or the “outcome” causes the “exposure"[33], and it is done using the SNPs to study the causal relationship in the separate GWASs [34]. The BMI genetic IV was obtained from a GWAS of UKB participants (https://gwas.mrcieu.ac.uk/; GWAS ID: ukb-b-19953). AD genetic IV data were also obtained from a GWAS [(https://gwas.mrcieu.ac.uk/; GWAS ID: ieu-b-2; data from Alzheimer Disease Genetics Consortium (ADGC), European Alzheimer's Disease Initiative (EADI)]. To remove IVs with linkage disequilibrium, SNPs were clumped for independence if they had a correlation of *r*^2^ > 0.001 [35]. Two methods—Inverse variance weighted (IVW)-MR and MR-Egger—have been primarily used for MR analysis [36]. To further ensure the reliability of the MR results, three other methods including Weighted median, Simple mode and Weighted mode were also used simultaneously.

First, we explored the causal relationship between BMI (exposure) and AD (outcome). The exposure and outcome data are available in the Additional file 2: Tables S2 and S3, respectively. After harmonizing the effect allele and removing the SNPs for being palindromic with intermediate allele frequencies, the remaining 433 SNPS were further analyzed using MR. After calculating the MR results, we next conducted a sensitivity analysis [37] mainly from the following three aspects: (1) Heterogeneity test: To test for differences among IVs. As these results showed a strong heterogeneity among IVs *(p* < 0.05), we next used a random effects model to estimate MR effect size. However, the random effects model yielded similar causal association results (*p* > 0.05). (2) Pleiotropy test: To test for horizontal pleiotropy in multiple IVs, which is often expressed by the intercept of MR Egger's law. Our results showed that there was no horizontal pleiotropy across multiple IVs (*p* > 0.05). (3) Leave-one-out sensitivity test: To calculate MR results of the remaining IVs after successive elimination of each IV. No matter which SNP was removed, it had no fundamental effect on the result, showing that our results were robust (Additional file 1: Figure S1). The visualization of the Mendelian randomization results and a detailed explanation of the conclusion are shown in Additional file 1: Figures S2–S4.

Second, we explored the reverse causal relationship between BMI (outcome) and AD (exposure). The exposure and outcome data are shown in Additional file 2: Tables S4 and T5, respectively. After calculating these MR results, we next performed a sensitivity analysis as previously described: (1) Heterogeneity test: These results showed a strong heterogeneity among IVs (*p* < 0.05), and a further random effects model yielded similar causal association results (*p* < 0.001). (2) Pleiotropy test: Our results showed that there was no horizontal pleiotropy across multiple IVs (*p* > 0.05). (3) Leave-one-out sensitivity test: Regardless of which SNP was removed, it had no fundamental effect on the results, indicating that our MR results were robust (Additional file 1: Figure S5). Meanwhile, the funnel figure showed that the funnel plot was symmetrical on the whole, without any obvious heterogeneity (Additional file 1: Figure S6).

R software (version 4.1.0) was used for all statistical analyses and the Two Sample MR package was used for MR analyses.

## Statistical analyses

Comparison of the baseline characteristics between control and AD groups was performed using the Chi-square (or univariate logistic regression) or Wilcoxon rank sum test. The *P* values were tested and adjusted by Benjamini–Hochberg false discovery rate (FDR) method. Continuous variables are represented as mean ± standard deviation or median ± interquartile range (IQR).

A restricted cubic spline (RCS) was used to further study the potential nonlinear relationship between BMI and AD. Moreover, an age subgroup analysis was also performed. The model was adjusted for age, Townsend deprivation index (TDI), sex, smoking, ethnicity, education level, alcohol use, hypertension, stroke, myocardial infarction, and diabetes. In addition, we divided BMI into three groups (< 23 kg/m^2^, 23–30 kg/m^2^, > 30 kg/m^2^) according to the RCS results.

A Kaplan–Meier survival curve was used to show the risk of AD among the three BMI groups (< 23 kg/m^2^, 23–30 kg/m^2^, > 30 kg/m^2^). The BMI (23–30 kg/m^2^) group was used as the control group, and the differences between the three groups were evaluated using log-rank tests.

A Cox proportional risk model was used to test the association between BMI and AD. The multivariable model 1 was unadjusted, model 2 was adjusted for age and sex, and model 3 was adjusted for age, TDI, sex, smoking, ethnicity, education level, alcohol use, hypertension, stroke, myocardial infarction, and diabetes.

A Cox proportional risk model was used to estimate the association between BMI and AD-GRS for risk of AD. The multivariable model was adjusted for age, TDI, sex, education level, alcohol use, hypertension, stroke, myocardial infarction, and diabetes.

All statistical analyses were performed with the R package (version 4.1.0). A *p* value < 0.05 was considered statistically significant.

## Results

### Basic characteristics of control and AD groups

The mean follow-up time was 11.58 years. We compared the basic characteristics of participants who developed AD (AD group, n = 886) and those who did not (control group, n = 474,927). As shown in Table 1, the AD group had a higher age and a larger TDI (*p* < 0.05). Those that were male, had a history of smoking, or were of mixed race as well as those with hypertension, diabetes, or a history of myocardial infarction or stroke had higher rates of AD (*p* < 0.05). Moreover, a lower education level correlated with a higher incidence of AD (*p* < 0.05). In addition, there were no significant differences in BMI between the AD and control groups.

**Table 1:** Comparison of the basic characteristics between the control (n = 474,927) and AD groups (n = 886)

Characteristics	Control group	AD group	P-adjusted
Age (Median, IQR)	58 (50,63)	66 (62,68)	< 0.001
TDI (Median, IQR)	−2.2 (−3.7,0.5)	−2 (−3.6,1.1)	0.0693
Sex (n, %)			< 0.001
Female	257,988 (54.3)	431 (48.6)	
Male	216,939 (45.7)	455 (51.4)	
Ethnicity (n, %)			< 0.01
White people	432,898 (91.2)	820 (92.6)	
Mixed people	17,394 (3.7)	40 (4.5)	
Other people	24,635 (5.2)	26 (2.9)	
Education (n, %)			< 0.001
College/University	155,879 (32.8)	168 (19)	
Other	319,048 (67.2)	718 (81)	
Smoking (n, %)			< 0.001
Never	260,085 (54.8)	422 (47.6)	
Previous	165,055 (34.8)	381 (43)	
Current	49,787 (10.5)	83 (9.4)	
Alcohol (n, %)			< 0.001
Never	20,271 (4.3)	77 (8.7)	
Previous	16,833 (3.5)	66 (7.4)	
Current	437,823 (92.2)	743 (83.9)	
Myocardial infarction (n, %)			
No	455,682 (95.9)	776 (87.6)	< 0.001
Yes	19,245 (4.1)	110 (12.4)	
Stroke (n, %)			< 0.001
No	462,880 (97.5)	787 (88.8)	
Yes	12,047 (2.5)	99 (11.2)	
Diabetes (n, %)			< 0.001
No	470,331 (99)	855 (96.5)	
Yes	4596 (1)	31 (3.5)	
Hypertension (n, %)			< 0.001
No	329,896 (69.5)	379 (42.8)	
Yes	145,031 (30.5)	507 (57.2)	
BMI (Median, IQR)	26.7 (24.1,29.9)	26.6 (23.9,29.4)	0.142

*IQR* interquartile range, *TDI* Townsend deprivation index

### The association between BMI and the risk of AD analyzed using RCS

**Fig. 2:**
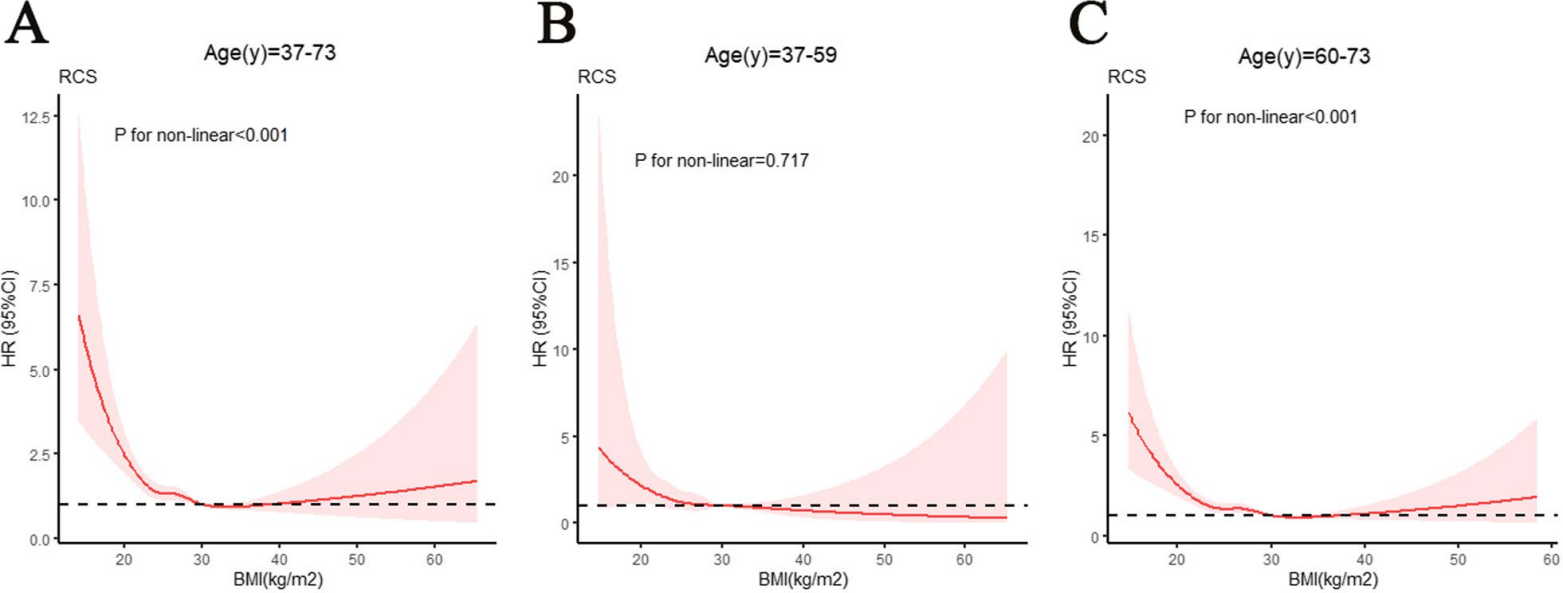
The restricted cubic splines (RCS) for analysis of the relationship between BMI and incidence of AD. **A**: 37–73 (years), **B**: 37–59 (years), **C**: 60–73 (years). Adjusted for age, TDI, sex, smoking, ethnicity, education level, alcohol use, hypertension, stroke, myocardial infarction, and diabetes

When assessing the results from previous studies, the effect of BMI on AD risk remains controversial [15–17]. As shown in Fig. 2A, there was a nonlinear relationship in the 37–73 years age group (*p* for non-linear < 0.001). Previous studies have defined 40–59 years as middle-aged and 60 years and older as elderly [38]. We further analyzed these age subgroups. Interestingly, as shown in Fig. 2B, there was no significant association between BMI and AD risk in the 37–59 years age group (*p* for non-linear = 0.717). However, as shown in Fig. 2C, there was a nonlinear relationship in the 60 years and older age group (*p* for non-linear < 0.001).

**Fig. 3:**
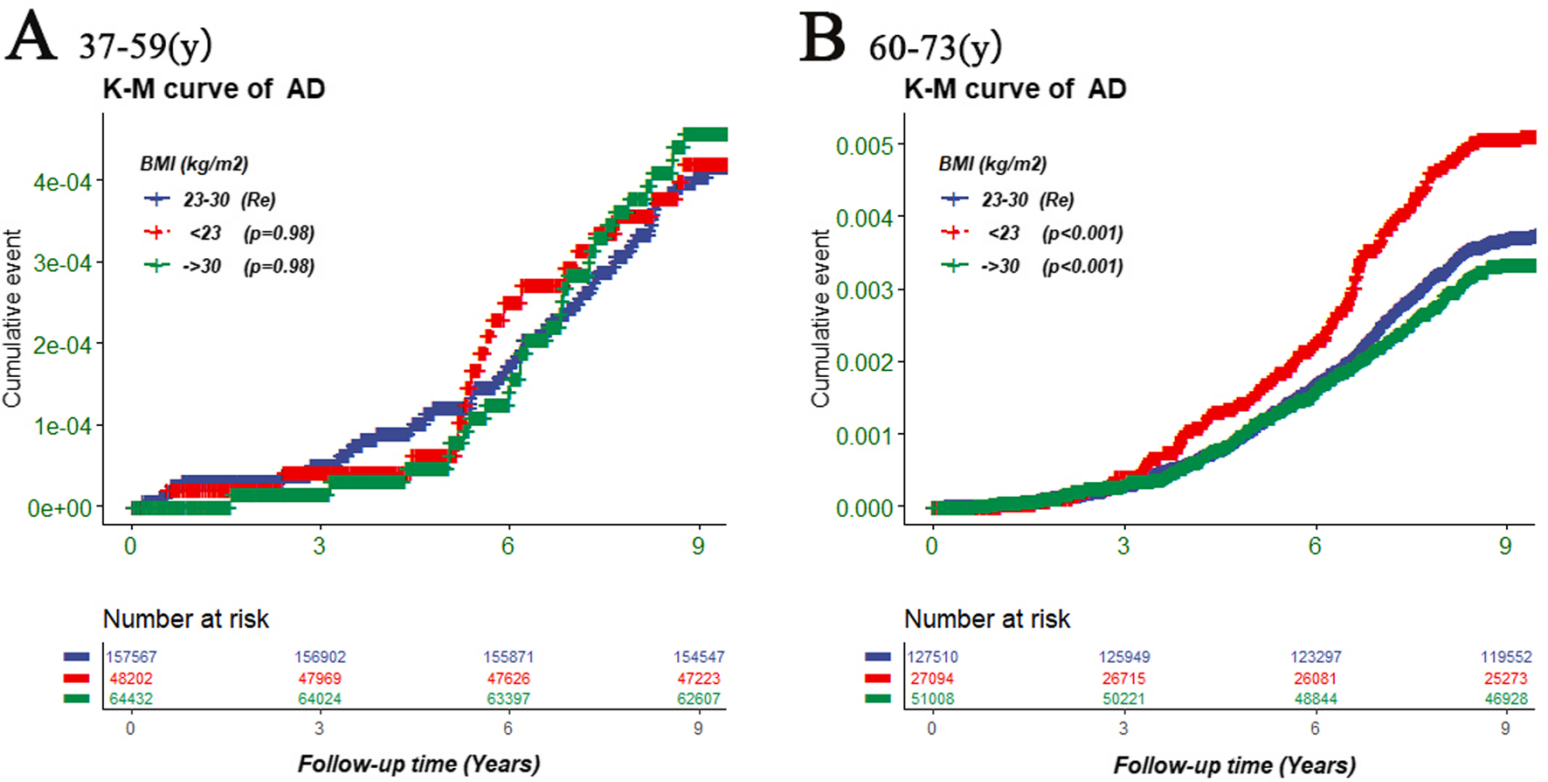
The Kaplan-Meier survival curve showing the risk of AD across the three BMI groups. **A**: 37–59 (years), **B**: 60–73 (years). The BMI (23–30 kg/m^2^) group was used as control group, and the differences between the three groups (< 23 kg/m^2^, 23–30 kg/m^2^, and > 30 kg/m^2^) were evaluated using log-rank tests

To further explore the appropriate BMI range between BMI and the risk of AD, we divided BMI into three groups according to the RCS results shown in Fig. 2. We used Kaplan–Meier survival curves to demonstrate the risk of AD among the three BMI groups (< 23 kg/m^2^, 23–30 kg/m^2^, and > 30 kg/m^2^). As shown in Fig. 3A, there was no significant difference in risk of developing AD across the three BMI groups of the 37–59 years old group. However, for participants aged 60 years and older, there was a significant difference across the three BMI groups (Fig. 3B). Compared with the BMI (23–30 kg/m^2^) group, the BMI (< 23 kg/m^2^) group had a significant association with a higher risk of AD (*p* < 0.001), while the BMI (> 30 kg/m^2^) group had an association with a lower AD risk (*p* = 0.17).

In summary, our results indicated a nonlinear relationship between BMI and AD risk in participants aged 60 years or older, but not in participants aged 37–59 years. Moreover, for participants aged 60 years and older, the BMI (< 23 kg/m^2^) group had a significant association with a higher risk of AD.

### Hazard ratio estimation for AD using Cox proportional risk model

**Fig. 4:**
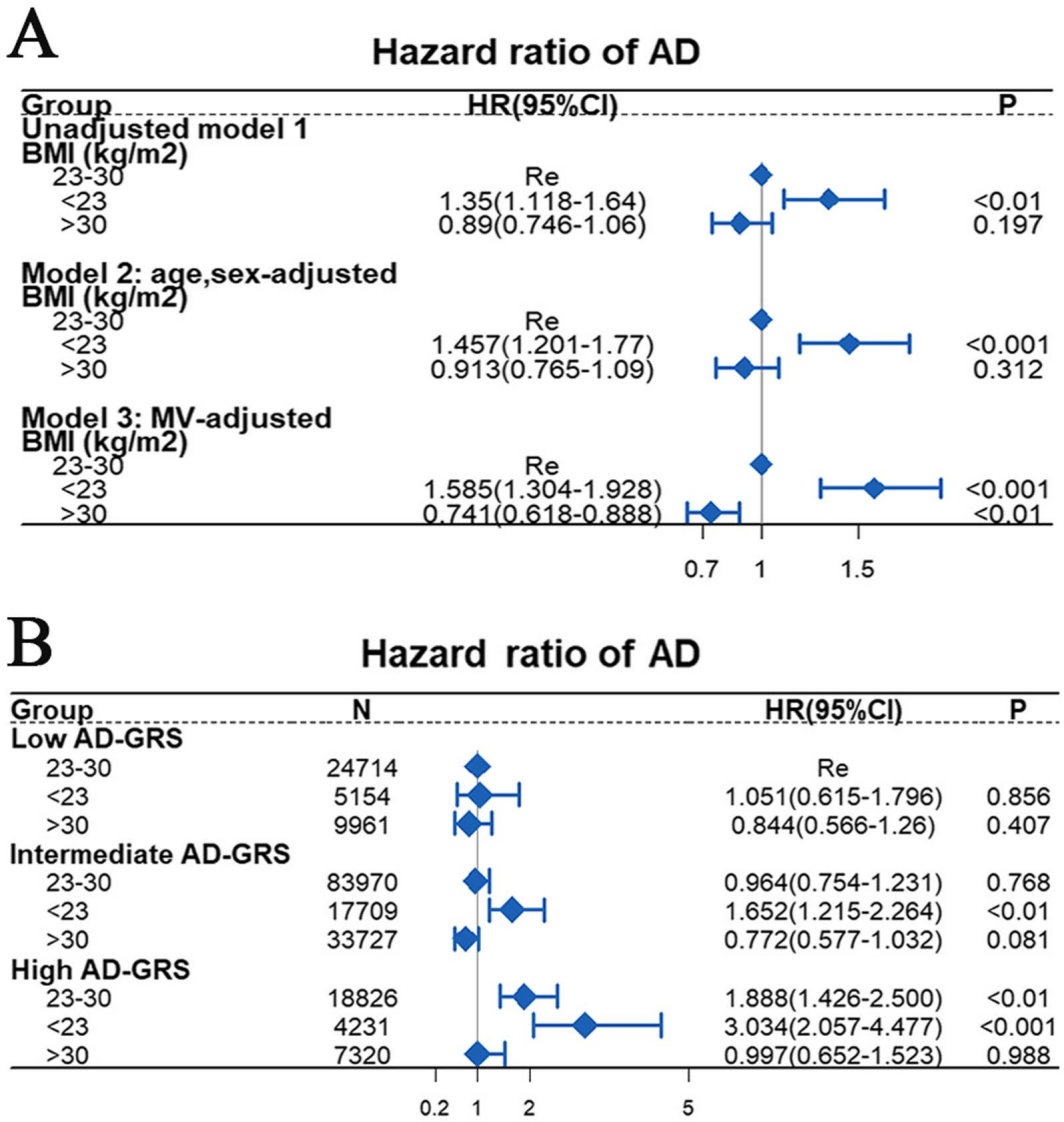
The Cox proportional risk model estimating the hazard ratio of AD. **A:** model 1 was unadjusted, model 2 was adjusted for age and sex, and model 3 was adjusted for age, TDI, sex, smoking, ethnicity, education level, alcohol use, hypertension, stroke, myocardial infarction, and diabetes. **B:** Joint association between long BMI and genetic risk score for AD. The multivariable model was adjusted for age, TDI, sex, education level, alcohol use, hypertension, stroke, myocardial infarction, and diabetes. The vertical line indicates a reference value of 1

As shown in Fig. 4A, for participants aged 60 years and older, compared with the BMI (23–30 kg/m^2^) group, the BMI (< 23 kg/m^2^) group was associated with a higher AD risk (HR = 1.35; 95% CI 1.118–1.64, *p* < 0.01) in the unadjusted model 1, the BMI (< 23 kg/m^2^) group was associated with a higher AD risk (HR = 1.585; 95% CI 1.304–1.928, *p* < 0.001) and the BMI (> 30 kg/m^2^) group was associated with a lower AD risk (HR = 0.741; 95% CI 0.618–0.888, *p* < 0.01) in the multivariable adjusted model 3.

For participants aged 60 years and older, our results indicated that the participants with the BMI (< 23 kg/m^2^) were associated with a higher AD risk.

### Joint association between BMI and AD-GRS for risk of AD

In combination, genetic and environmental factors have been considered main contributors to the progression of AD [3]. Therefore, we further investigated the potential interaction between genetic susceptibility and BMI on the risk of AD. As shown in Fig. 4B, there was no statistically significant interaction between BMI and GRS (*p* for interaction = 0.14). Additionally, participants with a combination of high AD-GRS and BMI (< 23 kg/m^2^) were associated with the highest AD risk (HR = 3.034; 95% CI 2.057–4.477, *p* < 0.001). In addition, compared with the BMI (< 23 kg/m^2^), the higher BMI was associated with lower risk of AD in participants with the same intermediate or high AD-GRS.

### Bidirectional MR of BMI and AD

**Fig. 5:**
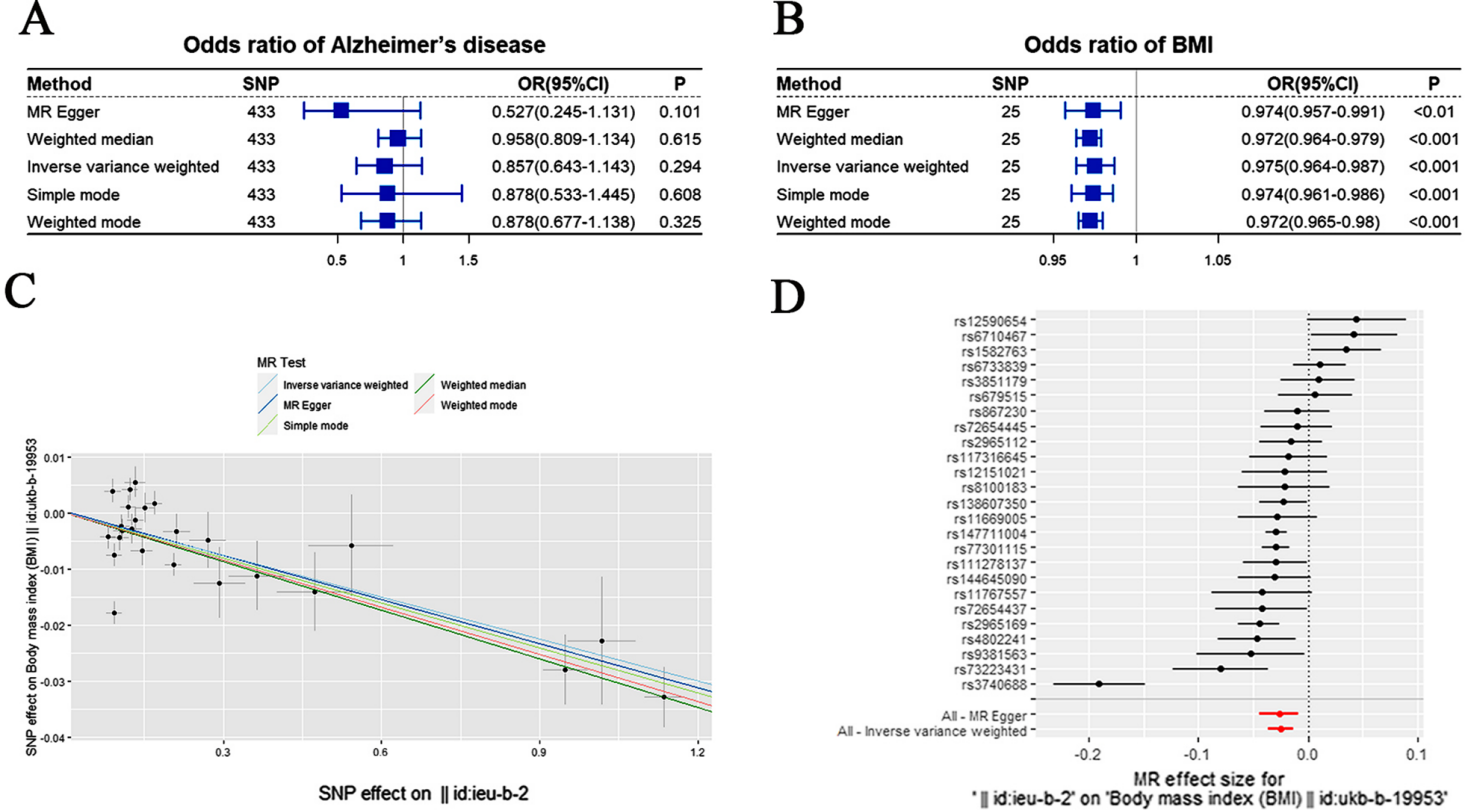
**A**: Summary of the Mendelian Randomization-Based Analysis of BMI (exposure) and AD (outcome). **B:** Summary of the Mendelian Randomization-Based Analysis of AD (exposure) and BMI (outcome).**C:** Scatter plot. Each point on this graph represents an IV, and the line on each point reflects a 95% confidence interval. **D:** The MR effect size for AD on BMI through the IVW and MR Egger methods

First, we explored the causal relationship between BMI (exposure) and AD (outcome). A summary of the MR-based analysis of BMI and AD risk is shown in Fig. 5A. Our five MR analysis results indicated that genetically predicted higher risk of BMI was not associated with the risk of AD (*p* > 0.05).

Second, we further explored the reverse causal relationship between BMI (outcome) and AD (exposure). The summary of the MR-based analysis of BMI and AD risk is shown in Fig. 5B. In the reverse direction, our five MR analysis results indicated that genetically predicted higher risk of AD was associated with lower BMI (OR < 1, *p* < 0.001). Figure 5C and D are visualizations of the MR results, which further confirmed the results shown in Fig. 5B.

Our results indicated there was a reverse causality between BMI and AD risk, and genetically predicted higher risk of AD was associated with lower BMI, suggesting that reduced BMI could be one of the early manifestations of AD.

## Discussion

In this large-scale study with an average follow-up time of more than 10 years, we have the following main findings: 1. our results indicated there was a nonlinear relationship between BMI and AD risk in participants aged 60 years or older, but not in participants aged 37–59 years; 2. for participants aged 60 years and older, our results indicated that participants with the BMI (< 23 kg/m^2^) were associated with a higher AD risk; 3. compared with the BMI (< 23 kg/m^2^), the higher BMI was associated with lower risk of AD in participants with the same intermediate or high AD-GRS; 4. there was a reverse causality between BMI and AD analyzed using bidirectional MR.

With global incidence of both obesity and dementia increasing year by year, understanding the causal relationship between BMI and AD risk has become a public health priority [39]. To the best of our knowledge, our study is the first to use bidirectional MR to establish a causal relationship between BMI and AD risk. Our results showed that there is a reverse causality between BMI and AD risk analyzed using bidirectional MR, suggesting that reduced BMI could be one of the early manifestations of AD.

Possible pathogenesis of BMI declines in AD patients has been investigated in previous studies. Reduced hippocampal volume and thinning of the entorhinal and medial temporal cortices are common imaging findings in AD patients [40]. Imaging data also indicate that brain structural changes, including changes of whole brain and hippocampal atrophy, are associated with alterations in body composition, including reductions in more specific measures of lean mass [41]. The potential mechanisms underlying the pathophysiological relationship between BMI and AD risk include neuropathological changes occur in regions like hypothalamus that play critical roles in regulation of energy metabolism and food intake [42]. Behavioral and cognitive changes associated with dementia can also affect weight by interfering with nutrition (forgetting to eat) or by reducing physical activity (a strong predictor of sarcopenia) [43]. In addition, Apolipoprotein E (*APOE*), produced primarily by astrocytes in the central nervous system, is a major cholesterol carrier that transports lipids to neurons to maintain synapses and promote damage repair, which is linked to increased accumulation of cortical amyloid-β (Aβ) [44]. The E4 allele of *APOE* gene (*APOE4*) is the strongest genetic risk factor for late AD [45]. The accumulation of Aβ in *APOE4* + individuals is regulated by leptin signaling in the hypothalamus [46], and leptin signaling pathway itself could lead to the synthesis and release of anorexia neuropeptides that may contribute to weight loss [47, 48].

Although our bidirectional MR results showed no positive causal relationship between BMI and AD risk due to the limitations of the MR method, a false-negative result cannot be ruled out. Our MR method mainly studied linear causality. However, our observational study found that the association between higher BMI and AD risk was non-linear. In addition, after the participants were grouped according to AD-GRS, a lower BMI was still associated with a higher risk of AD in the intermediate or high AD-GRS groups. Studies also showed that higher BMI-related genetic variants may slightly reduce the risk of AD [18], however, their non-linear relationship has been studied. Therefore, we speculated that there might be a non-linear causal relationship between BMI and the risk of AD. However, more research is needed to clarify this.

The possible mechanism underlying that high BMI is associated with lower risk of AD in older individuals remains poorly understood. Blautzik et al. showed that even among *APOE4* carriers, BMI was negatively associated with cortical amyloid load, glucose metabolism in posterior cingulate gyrus, and recent cognitive decline [47]. Adipose tissue releases molecules such as leptin and adiponectin, which bind to receptors in the hippocampus to regulate neuronal excitability, increase synaptogenesis, and prevent amyloid-induced neuronal cell death [49, 50]. In addition, microglia are innate immune cells of the central nervous system, which can prevent development of AD by inhibiting accumulation of Aβ [51]. Adiponectin can inhibit Aβ-induced inflammation and promote anti-inflammatory properties of microglia [52, 53]. Adiponectin receptor agonists can suppress microglia and astrocyte activation and restore microglia Aβ phagocytosis in mouse models of AD [54]. Since AD is an age-related disease, this might partially explain why high BMI in old age is associated with a lower risk of AD development [55]. However, further research will be needed to understand this mechanistic basis.

There is no significant association between BMI and AD risk in observational studies of participants aged 37–59 years old using RCS. Because the preclinical phase of dementia can last for more than 10 years [56], most of the participants may not have reached the diagnostic criteria for AD, and this population (37–59 years old) requires further follow-up in the future. Genetic and environmental factors have been considered contributors to the progression of AD [3]. To our knowledge, this is the first study to investigate the interaction between BMI and GRS on development of AD. As expected, for participants aged 60 and older, we observed that participants with high genetic risk had a higher risk of AD. In addition, a lower BMI (< 23 kg/m^2^) was associated with a higher risk of AD in the intermediate and high AD-GRS groups. Since genetic factor is an unmodifiable factor for the risk of AD, more attention should be paid to the management of BMI, especially in the populations with intermediate or high AD-GRS. It is considered that increased BMI (greater than 30 kg/m^2^) may lead to cardiovascular and metabolic diseases [57]. In addition, we also found that there was a U-shaped association between BMI and all-cause mortality, and higher BMI (BMI > 30 kg/m^2^) and lower BMI (BMI < 23 kg/m^2^) were associated with a higher risk of all-cause mortality (Additional file 1: Figure S7). These findings suggest that a higher BMI (BMI > 30 kg/m^2^) was not associated with a higher risk of AD, possibly due to the complications that had led to death in participants before AD was diagnosed, further validation is needed in future studies. Therefore, BMI (23–30 kg/m^2^) may be a potential intervention for AD without increasing complications and all-cause mortality.

## Conclusions

There was a reverse causality between BMI and AD risk analyzed using MR. For participants aged 60 and older, the higher BMI was associated with a lower risk of AD in participants with the same intermediate or high AD genetic risk. BMI (23–30 kg/m^2^) may be a potential intervention for AD.

## Limitations

This study was based on the UKB, which includes participants of a predominantly European ancestry. While this may affect the applicability of the results to other ethnicities, it does not change the internal validity of this study. During our follow-up, the BMI of participants may change, which is also one of the limitations of this study. However, the design of randomized trials for BMI is hardly feasible, and future longitudinal trajectory changes may overcome this limitation.

## Supplementary Information

**Additional file 1: Figure S1.** Leave-one-out sensitivity test (BMI served as the exposure and AD as outcome), no matter which SNP was removed, it had no fundamental effect on the result, and it showed that the MR result was robust. **Figure S2.** Scatter plot. Each point on this graph actually represents an IV, and the line on each point reflects the 95% confidence interval. The horizontal axis is the effect of SNP on exposure (BMI), the vertical axis is the effect of SNP on outcome (AD), and the colored line represents the MR fitting result. It suggested that exposure (BMI) had no positive causal relationship with outcome (AD). **Figure S3.** Forest map. MR effect size for AD on BMI. Each solid horizontal line in the forest map reflects the estimated result of a single SNP using the Wald ratio method. It indicated that the increased BMI did not significantly reduce the risk of AD by the IVW and MR Egger methods. **Figure S4.** Funnel figure. The funnel plot was symmetrical on the whole without obvious heterogeneity. **Figure S5.** Leave-one-out sensitivity test (AD served as the exposure and BMI as outcome), no matter which SNP was removed, it had no fundamental effect on the result, and it showed that the MR result was robust. **Figure S6. **Funnel figure. The funnel plot was symmetrical on the whole without obvious heterogeneity. **Figure S7. **A: Restricted cubic splines (RCS) for analysis of the relationship between BMI and incidence of AD. Adjusted for age, TDI, sex, smoking, ethnicity, education level, alcohol use, hypertension, stroke, myocardial infarction, and diabetes. B: Cox proportional risk model estimating the hazard ratio of AD. model 1 was unadjusted, model 2 was adjusted for age and sex, model 3 was adjusted for age, TDI, sex, smoking, ethnicity, education level, alcohol use, hypertension, stroke, myocardial infarction, and diabetes.

**Additional file 2: Table S1.** the single nucleotide polymorphisms (SNPs) that showed significant genome-wide association with AD,(the SNP loci of newly discovered genes in the UKB database were not included). **Table S2.** BMI served as the exposure (based on the UKB study , https://gwas.mrcieu.ac.uk/; GWAS ID: ukb-b-19953). **Table S3.** The datasets as AD outcome (based on another GWASs, https://gwas.mrcieu.ac.uk/; GWAS ID: ieu-b-2). **Table S4.** AD served as the exposure (based on another GWASs, https://gwas.mrcieu.ac.uk/; GWAS ID: ieu-b-2). **Table S5.** BMI served as the outcome (based on the UKB study , https://gwas.mrcieu.ac.uk/; GWAS ID: ukb-b-19953)

## Data Availability

The data that support the findings of this study are available from UK Biobank but restrictions may apply to the availability of these data, so they are not publicly available. However, the data are available from the authors with permission from the UK Biobank.

## Abbreviations

BMI: Body mass index
AD: Alzheimer's disease
AD-GRS: Alzheimer's disease genetic risk score
UKB: UK Biobank
MR: Mendelian randomization
SNPs: Single nucleotide polymorphisms
GWASs: Genome-wide association study
IV: Instrumental variable
RCS: Restricted cubic spline
TDI: Townsend deprivation index
