# Supplementary material for: Body mass index, genetic susceptibility, and Alzheimer's disease: a longitudinal study based on 475,813 participants from the UK Biobank
Source: J Transl Med. 2022 Sep 9;20:417. doi: 10.1186/s12967-022-03621-2 (PMC9463868; doi:10.1186/s12967-022-03621-2)
Supplement: Supplementary file 1 — Additional file 1: Figure S1. Leave-one-out sensitivity test (BMI served as the exposure and AD as outcome), no matter which SNP was removed, it had no fundamental effect on the result, and it showed that the MR result was robust. Figure S2. Scatter plot. Each point on this graph actually represents an IV, and the line on each point reflects the 95% confidence interval. The horizontal axis is the effect of SNP on exposure (BMI), the vertical axis is the effect of SNP on outcome (AD), and the colored line represents the MR fitting result. It suggested that exposure (BMI) had no positive causal relationship with outcome (AD). Figure S3. Forest map. MR effect size for AD on BMI. Each solid horizontal line in the forest map reflects the estimated result of a single SNP using the Wald ratio method. It indicated that the increased BMI did not significantly reduce the risk of AD by the IVW and MR Egger methods. Figure S4. Funnel figure. The funnel plot was symmetrical on the whole without obvious heterogeneity. Figure S5. Leave-one-out sensitivity test (AD served as the exposure and BMI as outcome), no matter which SNP was removed, it had no fundamental effect on the result, and it showed that the MR result was robust. Figure S6. Funnel figure. The funnel plot was symmetrical on the whole without obvious heterogeneity. Figure S7. A: Restricted cubic splines (RCS) for analysis of the relationship between BMI and incidence of AD. Adjusted for age, TDI, sex, smoking, ethnicity, education level, alcohol use, hypertension, stroke, myocardial infarction, and diabetes. B: Cox proportional risk model estimating the hazard ratio of AD. model 1 was unadjusted, model 2 was adjusted for age and sex, model 3 was adjusted for age, TDI, sex, smoking, ethnicity, education level, alcohol use, hypertension, stroke, myocardial infarction, and diabetes. [file 12967_2022_3621_MOESM1_ESM.docx]

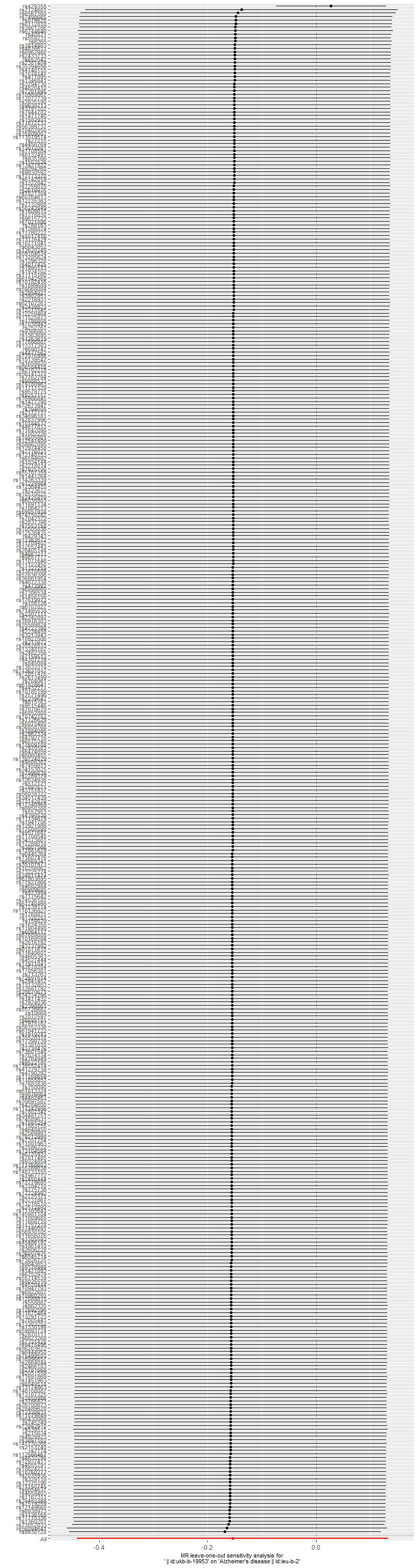


**Figure S1:** Leave-one-out sensitivity test (BMI served as the exposure and AD as outcome), no matter which SNP was removed, it had no fundamental effect on the result, and it showed that the MR result was robust.


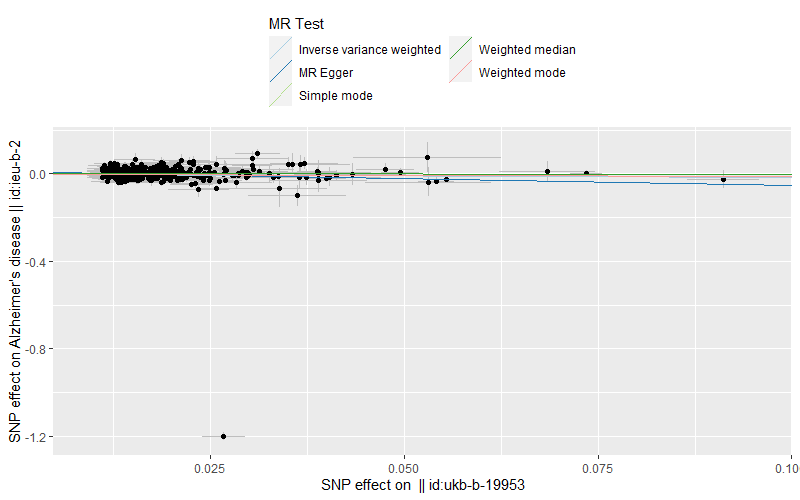


**Figure S2:** Scatter plot. Each point on this graph actually represents an IV, and the line on each point reflects the 95% confidence interval. The horizontal axis is the effect of SNP on exposure (BMI), the vertical axis is the effect of SNP on outcome (AD), and the colored line represents the MR fitting result. It suggested that exposure (BMI) had no positive causal relationship with outcome (AD).


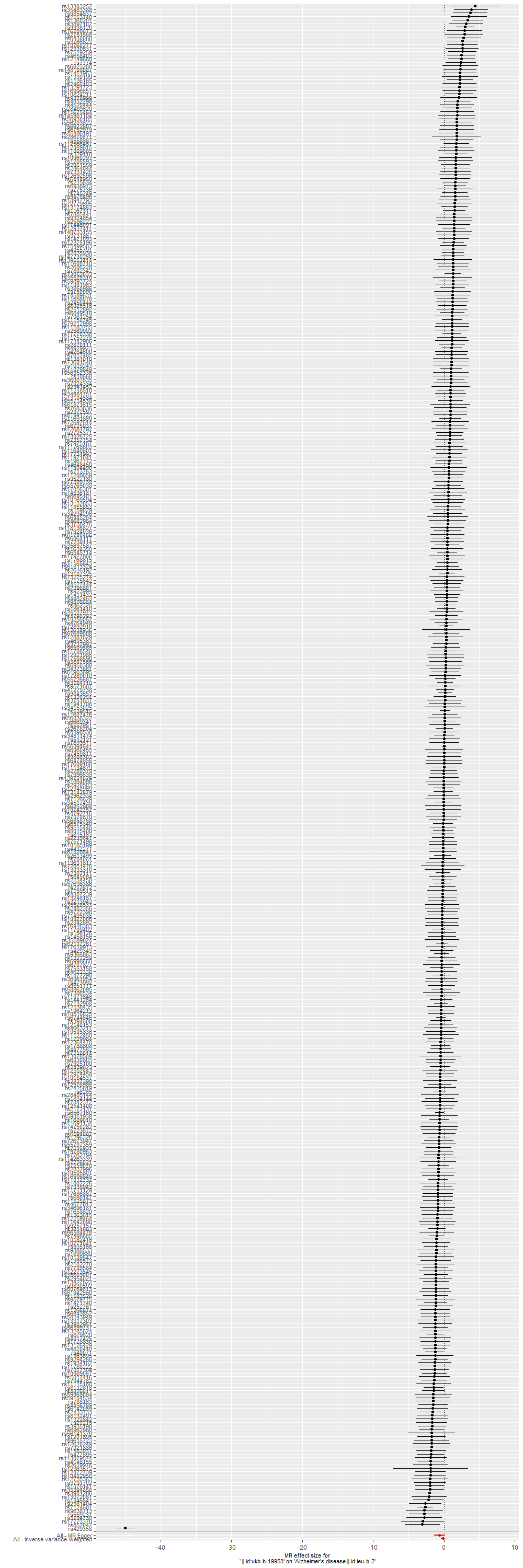


**Figure S3** Forest map. MR effect size for AD on BMI. Each solid horizontal line in the forest map reflects the estimated result of a single SNP using the Wald ratio method. It indicated that the increased BMI did not significantly reduce the risk of AD by the IVW and MR Egger methods.


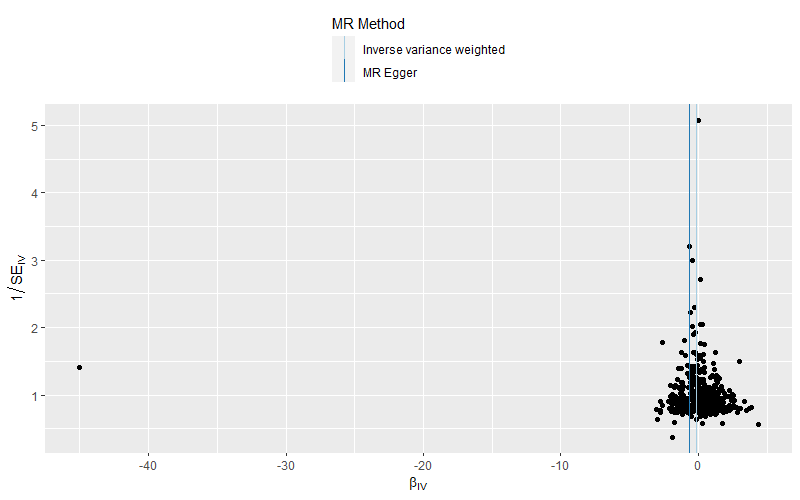


**Figure S4:** Funnel figure. The funnel plot was symmetrical on the whole without obvious heterogeneity


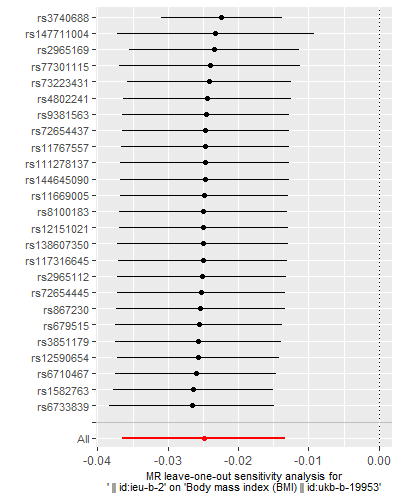


**Figure S5:** Leave-one-out sensitivity test (AD served as the exposure and BMI as outcome), no matter which SNP was removed, it had no fundamental effect on the result, and it showed that the MR result was robust.


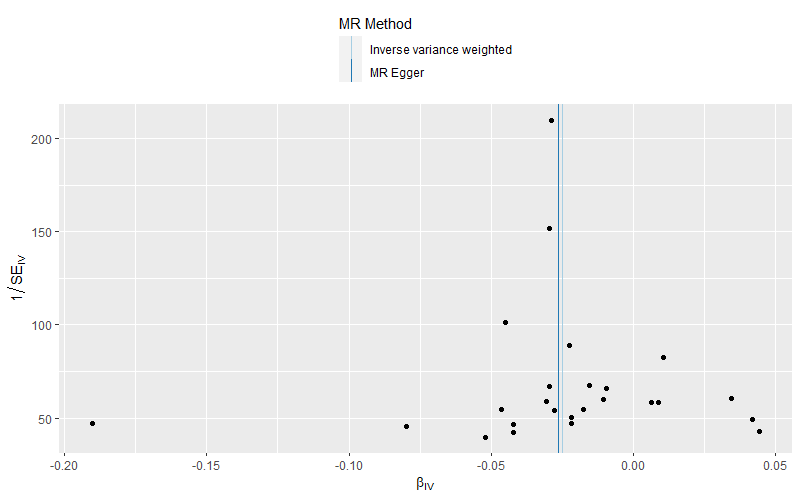


**Figure S6:** Funnel figure. The funnel plot was symmetrical on the whole without obvious heterogeneity


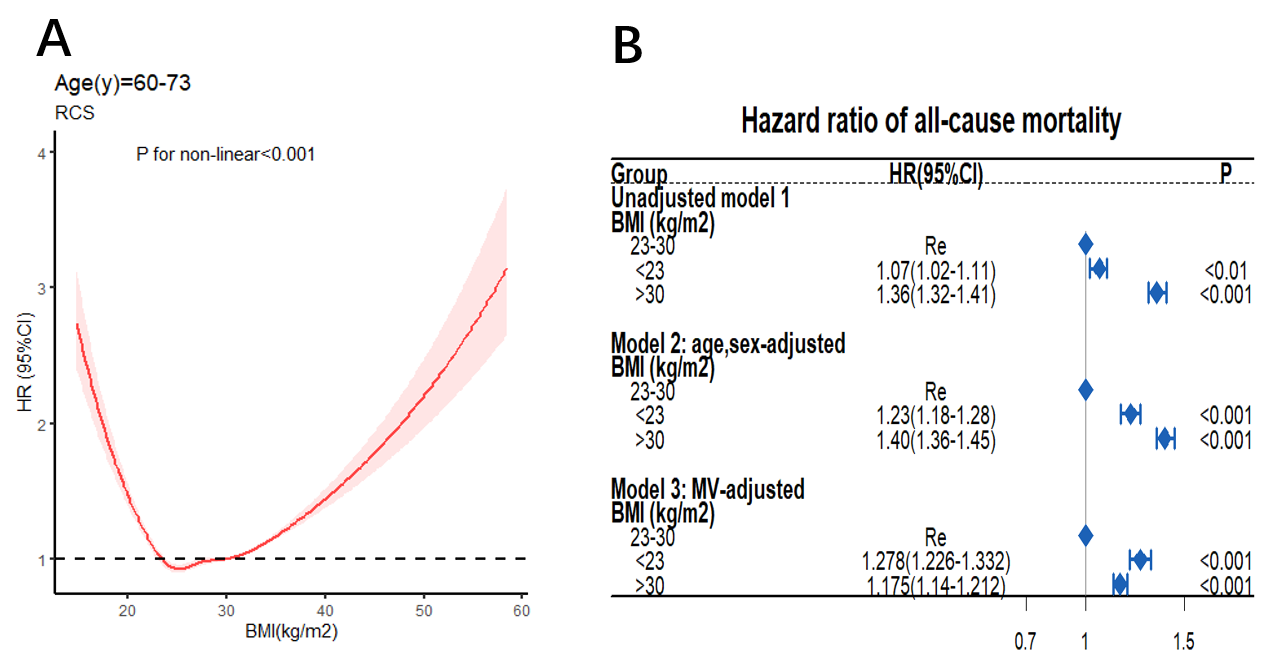


**Figure S7.** A: Restricted cubic splines (RCS) for analysis of the relationship between BMI and incidence of AD. Adjusted for age, TDI, sex, smoking, ethnicity, education level, alcohol use, hypertension, stroke, myocardial infarction, and diabetes. B: Cox proportional risk model estimating the hazard ratio of AD. model 1 was unadjusted, model 2 was adjusted for age and sex, model 3 was adjusted for age, TDI, sex, smoking, ethnicity, education level, alcohol use, hypertension, stroke, myocardial infarction, and diabetes.
